# Supplementary material for: Comparative Genomics of Mortierellaceae Provides Insights into Lipid Metabolism: Two Novel Types of Fatty Acid Synthase
Source: J Fungi (Basel). 2022 Aug 23;8(9):891. doi: 10.3390/jof8090891 (PMC9503022; doi:10.3390/jof8090891)
Supplement: Supplementary file 1 [file jof-08-00891-s001.zip › Supplementary Table S1.pdf]

Supplementary Table S1. The genes number of fatty acid metabolism in the 19 strains of Mortierellaceae

Species	HK	GAPDH	FAS	Delta5	Delta6	Delta9	Delta12	ELO2	ELOVL	ME	ACL	AMPD	ACC
A. wolfii NRRL 6351	4	1	1	1	1	1	1	1	1	3	2	1	1
D. globulifera REB-010B	6	1	2	1	1	2	1	1	2	4	2	2	1
M. alpina AD072	4	1	1	1	1	2	1	1	2	4	2	1	1
M. alpina AD071	4	1	2	1	1	3	1	1	2	4	2	1	1
M. alpina ATCC 32222	4	2	1	1	1	2	1	1	2	4	2	1	2
M. alpina B6842	4	2	1	2	1	3	1	1	2	4	2	1	1
M. alpina CCTCC M-207067	4	2	1	1	1	2	1	1	2	4	2	1	1
M. alpina CGMCC 20262	4	2	1	2	1	2	1	1	2	4	2	1	1
M. alpina CK1249	4	1	1	1	1	1	1	1	2	5	2	1	2
M. alpina GBA31	4	1	1	2	1	3	1	2	2	3	2	1	1
M. alpina LL118	4	2	1	1	0	2	1	1	2	4	2	1	1
M. alpina NRRL 66262	4	2	1	2	1	3	1	1	2	4	2	1	1
M. amoeboidea CBS 889.72	5	2	1	1	1	2	1	1	2	4	2	1	2
M. antarctica KOD 1030	5	1	2	1	1	1	1	2	1	5	1	1	1
M. elongata AG-77	5	2	1	1	1	4	1	1	1	4	2	2	1
M. epicladia AD058	4	2	1	1	1	1	1	2	1	5	2	1	2
M. gamsii NVP60	5	1	1	0	1	4	1	1	1	5	2	2	1
M. schmuckeri CGMCC 20261	3	2	1	1	1	4	1	1	1	4	2	2	1
M. verticillata NRRL 6337	4	2	1	1	1	4	1	2	2	6	2	1	1

Note: ACC: Acetyl-CoA carboxylase; ACL: ATP citrate (pro-S)-lyase; AMPD: AMP deaminase; Delta5: Delta-5 desaturase; Delta6: Delta-6 desaturase; Delta9: Delta-9 desaturase; Delta12: Delta-12 desaturase; ELO2: Fatty acid elongase 2; ELOVL: Elongation of very long chain fatty acids protein; FAS: Fatty acid synthase; GAPDH: Glyceraldehyde 3-phosphate dehydrogenase; HK: Hexokinase; ME: Malate dehydrogenase.
