# Supplementary figures and images for: Comparative Genomics of Mortierellaceae Provides Insights into Lipid Metabolism: Two Novel Types of Fatty Acid Synthase
Source: J Fungi (Basel). 2022 Aug 23;8(9):891. doi: 10.3390/jof8090891 (PMC9503022; doi:10.3390/jof8090891)

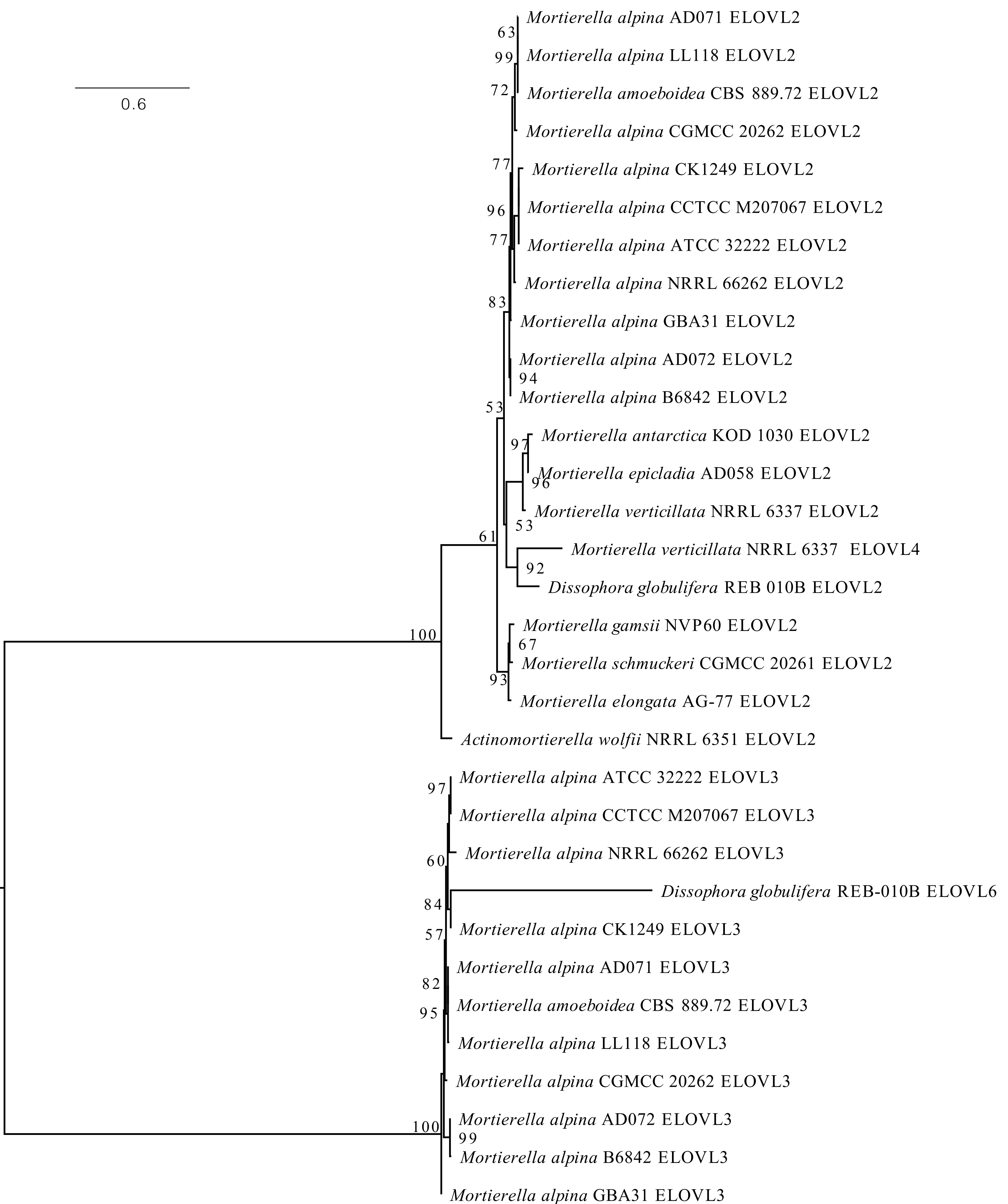

Supplement: Supplementary file 1 [file jof-08-00891-s001.zip › Supplementary Figure S1.pdf]

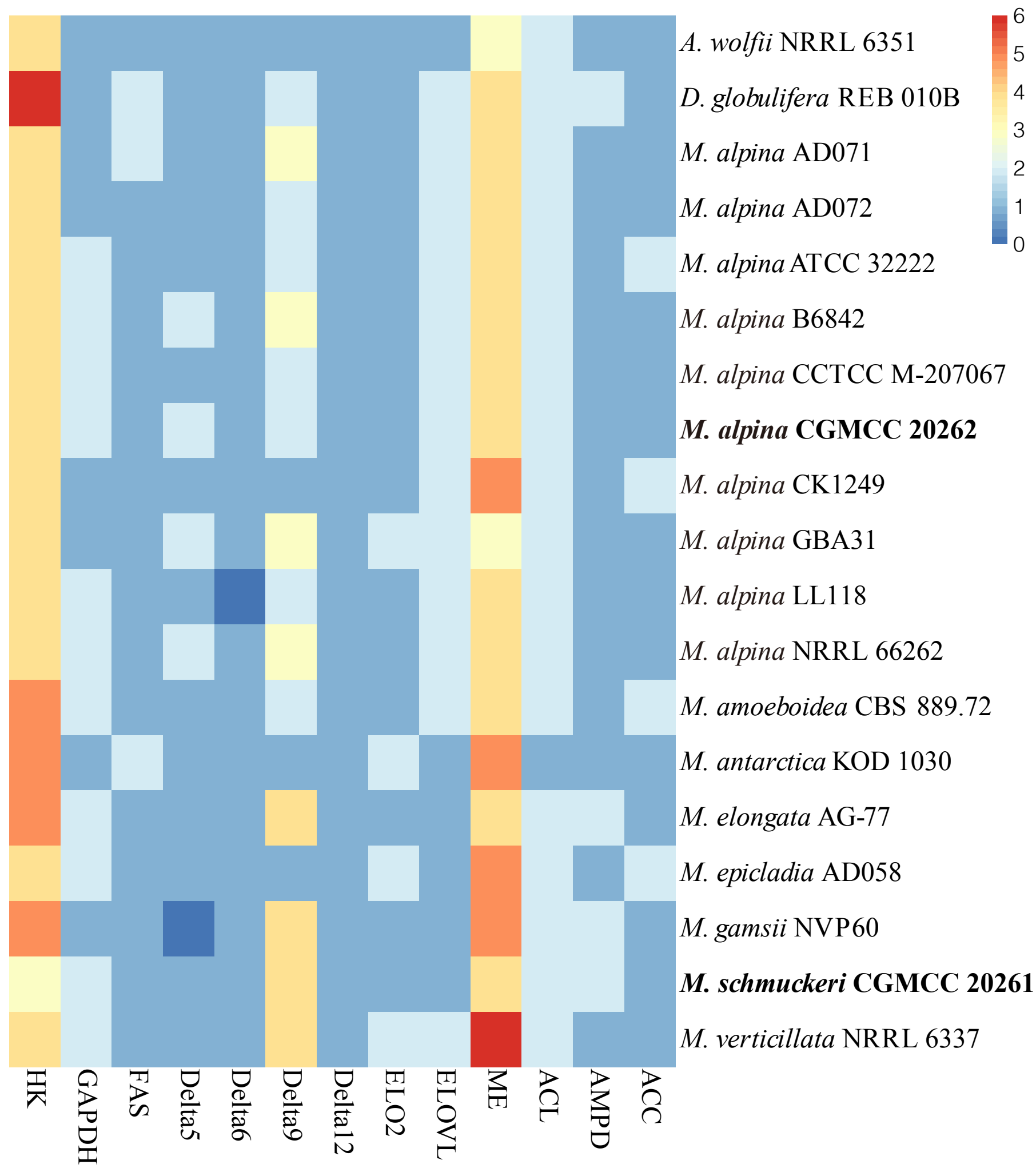

Supplement: Supplementary file 1 [file jof-08-00891-s001.zip › Supplementary Figure S2.pdf]
